# How to Avoid the Troubles Which Are Due to Uric Acid. II

**Published:** 1894-04-21

**Authors:** Alexander Haig


### Medical Progress and Hospital Clinics.

[*The Editor will be glad to receive offers of co-operation and contributions from members of the profession. All letters should be addressed to* The Editor, The Lodge, Porchester Square, London, W.]

### HOW TO AVOID THE TROUBLES WHICH ARE DUE TO URIC ACID.—II.

By Alexander Haig, M.A. and D.M. Oxon., F.R.C.P.

In a previous paper I mentioned a large number of troubles which I have been gradually led to attribute to excess of uric acid in the blood (uricacidæmia) or its concentration and irritating presence in certain fibrous tissues, and I now propose to consider our position with regard to the prevention and treatment of these troubles. As regards treatment there is only one indication, namely, to lessen the amount of uric acid in or passing through the body. Now I have calculated in the previous paper that of 11 grains of uric acid excreted 9 grains are formed in my body and 2 grains are introduced ready formed in my food, urea being 315 grains. Now the uric acid formed in my body bears to urea the relation of 1 to 35, and the uric acid introduced has the relation to urea of 1 to 157. I am far from saying that these proportions may not be modified by further knowledge, but they are near enough to illustrate my argument, and probably near enough for many practical clinical purposes.

Now supposing that a man comes to us who weighs 150 lbs. and excretes, as we find from examination of the urine on several occasions, about 4 grains of urea per pound (=600 grains), then this man will (on the above proportion) form about 17 grains a day of uric acid, and will introduce ready formed into his body about 4·0 grains; therefore he will have to excrete about 21·0 grains of uric acid, with a relation to urea of 1 to 28, if the output is to equal the income and formation—that is, if there is to be no accumulation of urates in his body. But this man who excretes 600 grains of urea a day will form and excrete also much more acid than a man who excretes only 300 to 400 grains; and if he excretes acid in the same proportion to urea that I do, namely, 1 grain of acid reckoned as oxalic acid for 6·1 grains of urea he will excrete 98 grains of acid a day, with this the total acidity of his urine will be greater, and the alkalinity of his blood less than my own. But the excretion of uric acid is inversely as the acidity of the urine, and directly as the alkalinity of the blood, therefore this man will not excrete uric acid in the relation to urea of 1 to 28, but more probably in the relation of 1 to 30. So that in place of excreting 21 grains of uric acid he may only excrete about 20 grains a day, and will retain in his body 1 grain every day, amounting to several hundred grains in a year.

Now, every man who has arrived at middle life, and for twenty years previously has been taking sufficient meat food to form urea at the rate of 4 grains per pound per day, more especially if he has been living a somewhat sedentary life (for exercise tends to keep down acidity), and has taken his fair share of wine and beer* has, I believe, probably accumulated in his body many hundreds of grains of uric acid, and is, therefore, in a position to suffer from gout, or from uricacidæmia, according to the other circumstances that affect his nutrition and metabolism. Let such a man meet with an accident and be laid on his back, his metabolism, urea and acidity, will run down, he will constantly excrete excess of uric acid from the stores in his body, and will suffer from all the effects of its excess in the blood (uricacidæmia), such as high blood pressure, headache, mental depression, general failure of nutrition, and eventually, if he is not able to recover from the injury and resume active life, Bright's disease. Or, again, let such a man take some unwonted exercise with perspiration which lowers his acidity and floods his blood with uric acid from his stores, and let him in this condition be exposed to cold and wet, which cause a sharp rise in acidity, the urate will be driven out of his blood into many joints, and he will have acute rheumatism; or let him, during his exertions, strain some ligaments or other joint structures, and then as his acidity rises again, after the exertion and perspiration are over, the urates will tend to accumulate round these injured structures, whose alkalinity is diminished, and he, will have acute gout.

Let such a man come to us with any of these troubles, say acute gout; we can tell him how he came to have an excess of urate in his body; we can tell him why he has got gout, and even why the particular joint is affected; and we can tell him exactly what he has to do to get rid of his gout and how to remain free from it. Let him reduce his income of animal food, so that his urea is 3 grains per pound instead of 4 grains, he will then excrete 450 grains of urea per day, will form 12·8 grains of uric acid, and will introduce 2·8 grains with his food. But his acidity will be lower, say 73 grains, and he will therefore possibly excrete the whole of the 15·6 grains of uric acic which he forms and introduces, so that he will cease to accumulate urates in his body, and will remain free from gout. In acute gout, therefore, it is merely necessary to clear out previous accumulations by means of a solvent, such as a salicylate, and then to alter the diet as above suggested to prevent reaccumulation.

If a patient comes to us suffering from excess of uric acid in his blood and its effects, this excess may be due partly to excessive introduction still going on from day to day (as in the wretched anæmic patients who think they are going to cure themselves by piling on meat, beef tea, &c., whereas I can actually cause anæmia in myself or anyone by means of these foods), or it may be, as in the aged and feeble, largely due to previous accumulations, which are being dissolved and swept out. If urea is too high, which under these conditions is not always the case, we must reduce it down to, say, three grains per pound per day, and must avoid all unnecessary introduction of urate by avoiding all foods which contain excess of it, such as beef tea, soup, strong tea, coffee, or cocoa, or such animal foods as lamb and veal, which appear to contain more than beef and mutton; or, again, liver or kidneys, which in animals, just as in man, contain more urates

* See "Uric Acid." London: J. & A. Churchill. Second Edition now in the press.

than other tissues, and this applies equally to all uric acid disease. Beyond thus reducing the income of urates two courses are open to us—we can clear the urate out of the blood at once, driving it into the liver, spleen, joints, and other fibrous tissues, and taking our chance of so producing gout or rheumatism, which not infrequently result from such treatment, or we can try to clear out all the urate accumulations by giving a course of solvents.

If we decide to clear the blood at once we can do this with mercury or iodides, or a combination of the two, as the ordinary iodide of mercury mixture, while iron, lead, and many other metals or their salts will act in the same way, and may precipitate an attack of gout or rheumatism, as they clear the blood of uric acid and relieve the high blood pressure and the headache, or the mental depression it is producing. If an attack of gout or rheumatism is thus produced, the best plan, probably, is to give a good course of salicylates, so as to sweep out as much as possible of the available urates, at the same time keeping up the acidity as much as possible so as to prevent the depressant effects which salicylates produce most markedly when the alkalinity of the blood is high, or when they are given (as I think they ought not to be) with alkalies.

I commonly allow such patients to take pretty freely of beer or any wine they may prefer, while they are on salicylates, as the acids of these beverages aid or at least do not hinder the solvent action of the drug, while they prevent to a large extent the depression it might otherwise cause.

We may thus, I believe, sum up the prevention and treatment of all uric acid disease in these three indications: 1. To lessen the amount of uric acid introduced into the body. 2. To lessen the amount of uric acid formed in the body. 3. To facilitate the excretion of uric acid, whether formed, introduced, or previously stored in the body. No. 1 is met by diminishing the income of animal food, which always contains uric acid ready formed, and avoiding, as far as possible, those animal and vegetable foods which are specially rich in uric acid or other members of the Xanthin group. No. 2 is met by diminishing the income of nitrogen; and in both these indications we must not act at random but take urea as our guide, as in the case sketched out above. No. 3 is partly met by the lowered acidity of the urine and the increased alkalinity of the blood which a diminution of animal and an increase of vegetable food produces; but where large amounts of urate have already accumulated in the body salicylate of soda may be given in the dose of gr. xv. to xx. ter. for four to six weeks in every two or three months for a year, eighteen months or more.

Where it is inconvenient or impossible to estimate urea, those articles of food which contain most uric acid or other members of the Xanthin group may be cut out, and if it still appears that the animal food consumed bears more than the proper physiological proportion to the vegetable food, it may be reduced to two-thirds or one-half of its previous quantity, watching the effect on the patient's weight and general condition; but where urea can be estimated, it should, in my opinion, never be neglected.

